# 

**Published:** 1868-01

**Authors:** 


### INDEX TO VOL. IX.

PAGE.

Abortion, 623, 666
Abyssinian Expedition, Mortality of the, 758
Academy of Medicine, New York, 229
Address on Medical Education by J. S. Jewell, M.D., 323
Addison's Disease. By A. Young, M.D., 5
Advertising, 187, 625
Albuminuria, Treatment of, 104
Alcohol, Abuse of in Medical Practice, 47
Anæsthesia, Apparent Exercise of Volition during Complete, 747
Anæsthesia, Monument to, 687
Apology, 752
Archives de Physiologie, 621
Archives de Physiologie, A Book, 375
Arteries, Ligation of, 100
Association, American Medical, 121, 190, 249, 350, 376, 444, 686
Association, Medical, Military Tract, 283
Association, Alumni, 52, 220, 249
Association, Missouri Medical, 120

Bartholow, Dr. Roberts, 55
Belladonna, Poisoning by, 97
" Detecting, 120
Bennet Eclectic Medical College, 564
Blood, Transfusion of, 506
Bloodvessels, On Some Results of a Retrograde Engorgement of, 728
Bottles, New Mode of Packing, 57
Bowels, A Book on Constipation of. By S. B. Birch, 685
Bromide of Ammonium in Dysmenorrhœa. By A. A. Dunn, M.D., 583

Caffein, Action of, 625
Calculi, Biliary. By R. Dunlap, M.D., 271
Calculi, Civiale's Collection, 40
Case, A Remarkable, 506
"Cerebration." By Henry M. Lilly, M.D., 705
Cerebration, Unconscious. By S. P. Breed, 449
Chloroform Poisoning, Position in. By E. L. Holmes, M.D., 538
Chloroform, Death from, 57, 253, 337, 698

PAGE.

Chloroform in Intermittent Fever. By D. Scott, M.D., 66
Chlorodyne, 117
Chlorosis, Blood-Corpuscles in, 185
Cholera in India, 190
Cholera. By John C. Peters, M.D., 286, 385, 513
Cholera, Report on. By N. S. Davis, M.D., 527
Cholera, Report on, 492
Cholera in 1867—*Resume*, 18
Chair in French Academy of Sciences, 120
Clinic at Mercy Hospital. By Professor N. S. Davis, 6, 337, 477, 595, 670, 748
Clinic on Epilepsy. By C. H. Jones, M.D., 599
Clinical Cases from Practice. By N. S. Davis, M.D., 71, 552
College, Medical, Chicago, 114, 186, 441, 562, 753
College, Medical, Detroit, 753
College, Medical, of South Carolina, 500
College, Medical, Changes, 564
College, Medical, Rush, 114
College, Medical, of Alabama, 526
College, Medical, Instruction, 495
College, Medical, Berkshire, 499
College, Medical, New, 499
College System, Medical, Changes in, 52
Colon, Length of the, in Young Children, 756
Commencements, Chicago Medical, 247
Compendium of Medical Science, 561
Condensation, 498
Contrast, A, 499
Consumption, Curability of, 630
Convulsions, A Case of. By S. T. Odell, M.D., 662
Convulsions, Infantile, 176
Correction by Dr. E. Andrews, 57
Correspondence by F. K. Bailey, M.D., 554
Correspondence from London, 482
Correspondence from F. B. Etheridge, M.D., 488

Deaths, 629
Deaths by Small-Pox, 189, 381
Decree of the Emperor of Russia, 699
Deposit, On the Nature of the

7980

PAGE.
Waxy, Lardaceous, or Amyloid. By William H. Dickinson, M.D., 742
Diagnosis, Physical, A Book on, 493
Digitalis, Effects of on Mind, 570
Discharges, Urethral. By J. S. Sherman, M.D., 535
Disease, Fungus Theory of, A Blow to the, 756
Diseases of Children, A Book on, 105, 621
Diseases of Women, A Book on, 246, 307, 622
Dissection of the Human Body, A Book on, 750
Doctor or Doctress? 752
Doctor's Bill, in reign of William III., 61
Druggists Renewing Prescriptions, 569

Eczema—Iodide of Lead, 312
Edinburgh Surgeon, Qualifications of an, in 1505, 758
Education, Medical, 108
Electro-Physiology, A Book, 308
Emigrants, Number of in 1867, 190
Embryotomy, Prof. Pagot on, 119
Endoscope, On the, 745
Endoscope, Improved Form. By E. Andrews, M.D., 468
Epilepsy, Nature of—A Case, 619
Epilepsy Treated by Strychnine, 302
Epistaxis, and Means of Arresting it, 506
Essay, Prize, on Physical Longevity, 59
Eye, Human, A Book on, 245, 561
Eye and Ear Clinic, Charitable, 566
Explanation, 186, 440
Extracts, Fluid, 311

Fevers, Mixed Types of, 290
Fistula, Rectal. By E. Andrews, M.D., 577
Fistula, Vesico-Vaginal, A Book on, 620
Force, Conservation of in Disease. By J. E. Hendricks, M.D., 550
Formulary, The Medical, 751
Fracture of the Leg, Splint for, 225

Gazette, Medical, of New York, 563
Give an Inch, and an Ell is taken, 115
Glanders, A Case of in the Human Subject. By W. O. Baldwin, M.D., 735
Glycerine, 623
Goff's Day-Book, Ledger, etc., 561

PAGE.
Gossypium as Emmenagogue, 569
Gonorrhœa—Starch Injections, 312
Graduates, Medical College, 310

Harelip, Operation for. By S. D. Mercer, M.D., 65
Health of London, 696
Heart Diseases, Treatment of, 107, 566
Hemorrhage after Extraction of Teeth, 758
Hemorrhage from Waxy Degeneration, 505
Hemorrhoids, Radical Cure of. By R. L. Walston, M.D., 3
Hernia, Operation for, without Opening the Sac, 759
Homœopathy, Modern, 474
Honors, 564
Hospital, Cook County, 565
Hotel for Invalids, 379
Hysteria, Curability of. By R. L. Walston, M.D., 710

Incompatibility of Iodide and Chlorate Potass., 116
Indigestions, A Book on, 491
Inebriation, A Short Paper on the Nature of, and the Means of Cure. By N. S. Davis, M.D., 711

Joints, Diseases of, A Book on, 308
Jacksonville Surgical Infirmary, 586, 624, 625
Journal, Louisville and Richmond Medical, 309
Just Published, 250

Klinik der Ohrenkrankheiten, A Book, 494
Knee, Anchylosis of. By J. S. Sherman, M.D., 580

Laughing Gas, Liquefying, 620
Lecture, Introductory. By Prof. N. S. Davis, 641
Lecture, Valedictory, 186
Lecture, Clinical, on Eye and Ear, 566, 629
Lecture, Valedictory. By Joseph S. Hildreth, M.D., 210
Letter from Vienna, 377
Letter from Paris, 79, 273, 278
Letter from London, 276
Letter by Dr. J. Dodge, 56
Life, Average Duration of in Italy, 253

PAGE.
Lithotomy and Lithotrity, ...... 60
Lungs and Throat, Treatment of, A Book on, ............... 50, 107

Marsden, Dr. William, of Quebec, 564
Materia Medica, A Book, ....... 309, 375, 492
Materia Medica, Therapeutic Value of Certain Remedies, ......... 234
Materia Medica, Synopsis of Lecture on, ..................... 50
Materia Medica, Dental, A Book on, ........................ 560
Medical Register and Directory of Philadelphia, ............. 655
Medicine, Science and Practice of, A Book on, .............. 622, 754
Medicine, Practical Report on. By S. P. Breed, M.D., ........ 193
Mercy Hospital, ................ 624
Metals, to Prevent from Rusting, . 115
Mortality among Physicians at Galveston, ................... 116
Mortality in 1867 in Chicago, ... 122
Monstra Abundantia. By A. H. DePuy, M.D., ............... 149
Mount Vernon Excursion, ...... 372

Nasal Therapeutics, ............ 693
Nélaton, M., ................. 633
Nerves, Division and Excision of, 695
Nerves, Effects of Traumatic Lesions of, .................... 744
Nerves of the Pia Mater, ........ 184
Neuralgia, Ovarian Treatment of, 651
Neuralgia, Treatment of. By N. S. Davis, M.D., .............. 6
Neuroses of the Skin, A Book on, 492

Obstetrics, A Book on, .. 105, 245, 560
Odontalgia, A Book on, ........ 375
Ophthalmoscope in Diseases of Nervous System, ............. 692
Otorrhœa, New Treatment of, ... 41
Ovariotomy, Treatment of, ...... 32
Oxygen, Inhalation after Tracheotomy, ..................... 684
Oxygen Mixture. By E. Andrews, M.D., ................ 656

Paralysis, Pathology of, ......... 614
Paralysis of One Leg. By M. M. Eaton, M.D., ................ 1
Paralysis, Treatment of, ........ 680
Paris Medical Faculty, ......... 699
Pathology, General, A Work on, . 308
Pepsin in Infantile Diarrhœa, ... 694

PAGE.
Periscope, ...................... 608
Pericardium, Calcareous Degeneration of. By Curtis F. Fenn, M.D., ...................... 321
Personal—Prof. W. H. Byford, .. 115
Pharmacy, Chicago College of, ... 221
Phthisis Pulmonalis, .... 309, 317, 490
Physicians, To, .... 116, 189, 252, 629
Physiology, Outlines of, Human and Comparative, A Book on, . 750
Plague, Cattle, Prevention of, ... 570
Placenta Prævia. By F. K. Bailey, M.D., ...................... 497
Plastic Surgery, A Book on, ..... 106
Polypi, Uterine, Treatment of. By M. M. Eaton, M.D., .......... 652
Polypus, Uterine. By S. P. Breed, M.D., ...................... 206
Practice, Notes on General. By John Forman, M.D., ......... 548
Pregnancy, Signs and Diseases of, 107
Pregnancy, Sickness of, ......... 499
Premiums, Fiske Fund, ......... 632
Principles and Practice of Medicine, A Book on, ............ 750
Prize, O'Reilly, ................ 444
Prizes, Boylston Medical, ....... 632
Probe, Bullet Musical, .......... 569
Profs. Pancoast and Gross, Public Reception to, ............... 757
Prof. Peaslee, Dartmouth College of, ......................... 759
Professors, .................... 499
Pyrosis, Sulphurous Acid in the Treatment of, ............... 747
Pus, Formation of by Inflammation, ...................... 48
Pyrethrum for Scabies, ......... 756

Record, Pocket, for Physicians, .. 187
Receipts of Money, 57, 116, 190, 252, 379, 441, 502, 565, 629 759
Rectal Bougie. By J. S. Sherman, M.D., ...................... 585
Remedies, Concentrate. By F. R. Payne, M.D., ................ 543
Report, Mortality, 58, 188, 251, 380, 442, 501, 567, 627, 691, 754
Reprint, Medical, The Monthly, .. 500
Reply to Dr. Hendricks' Review. By Z. C. McElroy, M.D., ..... 411
Report on Sanitary Condition of Chicago. By N. S. Davis, M.D., 257
Report of Board of Health, ..... 313
Report of Physician to Washingtonian Home, .............. 68
Reports of Pennsylvania Hospital, 106

PAGE.

Report of Epidemic Diseases of Illinois, 51
Resections, 118
Revaccination, Necessity of, 118
Review of Dr. McElroy's Hypothesis of Pathology of Typhoid Fever. By J. E. Hendricks, M.D., 269
Rheumatism, Muscular. By F. B. E., M.D., 593
Rheumatism, Alcoholic, 505
Richards, Dr. R. T., 443
Rickards' and Rigner's Experiments, 61
Rosin-Weed, 44

Scabies, Pyrethrum for, 756
Sciences, Medical, Half-Yearly Abstract of, 500
Shock, A Book on, 50
Society, Medical, Morgan County, 12, 150, 154, 346, 420, 423, 667
Society, Illinois State Medical, 114, 186, 249, 376, 426, 624
Society, Chicago Medical, 112, 280, 282, 341, 343, 345, 416
Soçiety, Chicago Medical, Proceedings of, 721
Society, Clark County Medical, 16, 557
Society, San Francisco Medical, 377
Society, Medical, of Nebraska, 564
Society, Medical, Champaign Co. 285
Society, Medical, Adams County, 75
Society, Medical, Meetings, 309
Sodæ Sulphis and Hyposulphis.
Spain, Statistics of, 757
By W. H. Prince, M.D., 398
Specimens, Anatomical, Preserved, 182
Speculum. By N. Bozeman, M.D., 156, 187
Spermatorrhœa, A Work on, 108
Spiders, 119
Spleen, Enlarged, Cured. By A. Given, M.D., 146
Spleen, Function of. By H K. Jones, M.D., 129
Stricture in Urethra, Treatment,
Subscribers, To 753
Sulphites in Zymotic Diseases. By P. G. Kelsey, M.D., 264
Sulphites in Fevers. By A. C. Simonton, M.D., 414
Surgery, Sr all Things in. By E. Andrews, M.D., 335
Sunstroke, Deaths from, 570
Surgery, Conservative. By A. S. Walter, M.D., 685

PAGE.

Surgery, Orthopœdic, A Book on, 246
Susceptibllity, Emotional. By T. Griffin, M.D., 592
Sydenham Society's Publications, 565
Syphilis, Early History of, 698

Tacile Corpuscles, The Structure of, Demonstrated, 758
Tænia, A New Treatment for, 61
Teaching, Summer, 310
Teeth, Extraction of, A Book on, 560
Therapeutics, Mechanical, 51
Thermometer, Clinical, 617
Thermometer in Typhoid Fever, 503
Tobacco, Causes Bald Heads and Gray Hairs, 697
Tubercular Matter, Inoculating upon Plants, 757
Turpentine as an Antidote to Phosphorus, 759
Typhoid Fever. By Z. C. McElroy, M.D., 139
Typhoid State in Different Diseases, 169

University, Cumberland, Medical Department of, 625
University of Nashville, 500, 562
University of Michigan, 310, 499, 565
University, Louisville, Medical Department of, 500
Uterine Affections, Treatment of by Arsenic, 682
Uterine Injections, 569
Uteri, Artificial, Procidentia of, 89
Uterus, Inversion, New Mode of Reduction, 87

Vaccination, Hand-Book of. By E. C. Seaton, M.D., 685
Venereal Diseases, Atlas of, 307, 493, 621, 751
Virchow's Personal Appearance, 697
Virus, Vaccine, Nature of, 502
Visiting List, Physician's, 634

Whalebone in Uterus. By M. M. Eaton, M.D., 218
Whooping-Cough. By John C. Peters, M.D., 674
Witnesses, Rights of Medical Experts as, 743
Women, A Hint for, 441
Womb, Rupture of. By S. A. McWilliams, A.M., M.D., 581

Young, Dr. D. S., 120

